# Effect of Protracted Free-Choice Chlortetracycline-Medicated Mineral for Anaplasmosis Control on *Escherichia coli* Chlortetracycline Resistance Profile from Pastured Beef Cattle

**DOI:** 10.3390/microorganisms9122495

**Published:** 2021-12-02

**Authors:** Alyssa R. Toillion, Emily J. Reppert, Raghavendra G. Amachawadi, K. C. Olson, Johann F. Coetzee, Qing Kang, Kathryn E. Reif

**Affiliations:** 1 Department of Diagnostic Medicine/Pathobiology, Kansas State University, Manhattan, KS 66506, USA; toillion@vet.k-state.edu; 2 Department of Clinical Sciences, Kansas State University, Manhattan, KS 66506, USA; erepper@vet.k-state.edu (E.J.R.); agraghav@vet.k-state.edu (R.G.A.); 3 Department of Animal Sciences and Industry, Kansas State University, Manhattan, KS 66506, USA; kcolson@ksu.edu; 4 Department of Anatomy and Physiology, Kansas State University, Manhattan, KS 66506, USA; jcoetzee@vet.k-state.edu; 5 Department of Statistics, Kansas State University, Manhattan, KS 66506, USA; qkang@k-state.edu

**Keywords:** *Anaplasma marginale*, AMR, antimicrobial resistance, CTC, *E. coli*

## Abstract

Anaplasmosis is an economically-significant, hemolytic, tick-borne disease of cattle caused by *Anaplasma marginale* which can cause clinical anemia and death. Current control options are limited, and FDA-approved antimicrobial control options do not have a defined duration of use. A practical and routinely used anaplasmosis control method involves feeding free-choice chlortetracycline (CTC)-medicated mineral to pastured cattle for several months. Constant antimicrobial use poses the risk of expediting the development and dissemination of antimicrobial resistance in off-target commensal bacteria in the bovine gastrointestinal tract. The objective of this study was to determine the CTC-susceptibility of *Escherichia coli* isolated from anaplasmosis endemic beef cattle herds provided different FDA-approved free-choice CTC-medicated mineral formulations, all intended to provide cattle a dosage of 0.5 to 2.0 mg CTC/lb bodyweight per day. A closed-herd, comprised of Hereford-Angus cows, naturally endemic for anaplasmosis, were grazed in five different pastures with one herd serving as an untreated control group. The other cattle herds were randomly assigned one of four FDA-approved CTC-medicated mineral formulations (700, 5000, 6000, and 8000 g CTC/ton) labeled for “the control of active anaplasmosis” and provided their respective CTC-medicated mineral formulation for five consecutive months. Fecal samples were collected monthly from a subset of cows (*n* = 6 or 10) per pasture. Fecal samples were cultured for *E. coli* isolates and the minimal inhibitory concentration of CTC was determined. Baseline CTC-susceptibility of *E. coli* was variable among all treatment and control groups. The susceptibility of *E. coli* isolates was significantly different between study herds over the treatment period (*p* = 0.0037 across time and 0.009 at the final sampling time). The interaction between study herds and treatment period was not significant (*p* = 0.075).

## 1. Introduction

The use of medically important antimicrobials in food-producing animals has raised concerns about promoting antimicrobial resistance (AMR) [1]. In 2019, the total amount of antimicrobial drugs actively marketed was approximately 11.4 million kilograms [2]. Of the total drugs marketed, 6 million kilograms were medically important antimicrobial drugs sold and distributed for food-producing animals, of which 2.5 million kilograms were for use in cattle [2]. Tetracycline antimicrobials are considered highly important and have consistently been the largest portion (67%) of all antimicrobials sold in the U.S. for food-producing animals, including in cattle [2]. There is strong evidence that antimicrobial-resistant bacteria can be transferred from livestock to humans [3,4]. A systematic literature review reported that reducing antibiotic use in food-producing animals decreased the prevalence of antibiotic-resistant bacteria in animals by about 15% and multidrug-resistant bacteria by 24–32% [5]. In many places, antibiotics are overused and misused in animals and people, and often given without professional oversight [6,7]. With a limited number of antimicrobials available, improper use can promote AMR and reduce the effective lifespan of the antimicrobial. As a means to reduce AMR in the U.S., all in-feed medically important antimicrobial drugs for use in food-producing animals have recently been put under veterinary oversight [8]. Despite an increased regulatory structure, some currently approved in-feed antimicrobial indications for food-producing animals do not have a defined duration of use. For example, chlortetracycline (CTC) indicated for “control of active anaplasmosis” does not have a current limit on the duration of use as long as a producer has a valid Veterinary Feed Directive (VFD).

Bovine anaplasmosis (hereafter referred to simply as anaplasmosis) is a globally occurring tick-borne disease of cattle [9]. Clinical disease is most commonly observed in mature cattle. Anaplasmosis is a disease of economic importance in the U.S., conservatively estimated to cost the U.S. cattle industry greater than $300 million per year [10,11]. The causative agent, *Anaplasma marginale*, invades and colonizes red blood cells (RBCs); mass destruction of infected RBCs can lead to anemia, the hallmark of clinical anaplasmosis. Other clinical anaplasmosis signs include icterus, lethargy, fever, aggression, abortions, and death. Infected cattle may never show signs of disease, especially if infected when young, and most animals (treated or untreated) will recover from disease and serve as infection reservoirs. Subsequent transmission events can occur via ticks, blood-contaminated biting fly mouthparts, or blood-contaminated equipment such as needles, dehorning, and castration equipment. Increased cattle movement has facilitated the spread of anaplasmosis into almost every continental state. In a Kansas seroprevalence study (*n* = 925 herds), 52.54% (486/925) of sampled cow-calf herds tested seropositive for *A. marginale* [12]. In other previous U.S. anaplasmosis seroprevalence studies, cELISA testing from a 2013–2014 slaughter survey (*n* = 215) in Mississippi found a seroprevalence of 29.02% (95% CI: 22.74–36.07%) [13]. In Texas, results from an active slaughter survey (*n* = 215) performed between August and December 2014 as well as reviewing Texas A&M Veterinary Medical Diagnostic Laboratory records of specimens submitted for anaplasmosis testing from January 2002 to June 2012 (*n* = 15,460) found the estimated seroprevalence of anaplasmosis in Texas to be 15.91% (95% CI: 15.34–16.50%) [14]. Samples taken from a Georgia (*n* = 293) auction barn and abattoir from 2013–2014 found the estimated seroprevalence to be 4.44% (95% CI: 2.61–7.44%) [15]. 

In beef production, antimicrobials are important to maintain or improve animal health towards increasing productivity and economic viability [16,17]. The most common antimicrobial-based anaplasmosis control for pastured cattle is CTC delivered in-feed or via medicated mineral. Currently, the FDA allows producers to provide CTC-medicated feed products for anaplasmosis control with no limits on the duration of use as long as the producer maintains a valid VFD [8]. In-feed or mineral supplementation of CTC is indicated for “control of active anaplasmosis” and can be administered free-choice or hand-fed. “Hand-fed” CTC-medicated feeds are provided and consumed daily (0.5 mg CTC/lb of BW daily) whereas “free-choice” CTC-medicated feeds are kept constantly available to the animal and intend to provide 0.5 to 2.0 mg CTC/lb of BW daily. The free-choice feeding method presumes that the animal will balance its own diet based on individual nutritional needs. There are two public (6000 g CTC/ton) and three proprietary (700, 5000, 8000 g CTC/ton) FDA-approved free-choice CTC-medicated feed formulations indicated for the control of anaplasmosis. All of these formulations are intended to deliver a dosage of CTC that will fall within the approved range when consumed in a free-choice manner (0.5 to 2.0 mg CTC/lb body weight/day). In a statewide survey of Kansas cattle producers, CTC-medicated feed products were used by 25.3% (109/431) of respondents, of which 76.1% (83/109) reported year-round use and only 23.9% (26/109) reported CTC use in the spring and summer months [12]. 

As antimicrobials have broad activity against multiple bacterial species, the use of antimicrobials to control one disease (i.e., CTC to control anaplasmosis) may have unintended consequences on other microbial community members (i.e., AMR development in off-target microbes such as *Escherichia coli*). Fecal shedding of resistant *E. coli* is common in cattle and is a public health concern due to the risk of foodborne transmission that can result in severe, or even fatal, disease in people [18]. Tetracycline resistance among *E. coli* in cattle is relatively common [19,20,21,22]. Previous studies demonstrated that exposure to in-feed CTC was associated with a temporary increase in the likelihood of recovering resistant bacteria [23,24]. In feedlot cattle, in-feed CTC administration (10 mg CTC/lb of body weight/day) for 5-days temporarily increased fecal tetracycline-resistant *E. coli* but did not impact long-term resistance in the *E. coli* population [19]. In a 314-day study where feedlot steers received CTC as a top-dress (11 ppm Aureomycin), 47.1% (3413/7184) of the *E. coli* isolates were found to be resistant [25]. Long-term use of medically-important antimicrobials such as CTC for anaplasmosis control may contribute to expediting the development of CTC-resistant bacteria in off-target commensal bacteria species (i.e., *E. coli)* in the bovine gastrointestinal tract. Therefore, the objective of this study was to determine the CTC susceptibility of *E. coli* isolated from anaplasmosis endemic beef cattle herds provided different FDA-approved free-choice CTC-medicated mineral formulations in a pasture setting. Regardless of CTC-medicated mineral formulation, the susceptibly profile of *E. coli* was hypothesized to decrease as treatment length increased, with little to no difference among approved CTC formulations intended to provide the same CTC dosages. Critical evaluation of antimicrobial treatment outcomes is not only important for the intended microbe target but off-target susceptible microbes as well. Understanding off-target implications of broad-acting drugs are important when developing drug use protocols and policy to both protect drug effectiveness and minimize broader unintended impacts. 

## 2. Materials and Methods

The Kansas State University Institutional Animal Care and Use Committee reviewed and approved all animal handling and animal care practices used in this study (IACUC #: 3858.1). All procedures were conducted in accordance with the Guide for the Care and Use of Animals in Agricultural Research and Teaching [26]. 

### 2.1. Experimental Design

#### 2.1.1. Study Herd 

A portion of the Kansas State University Cow-Calf herd (*n* = 245), a closed-herd, comprised of Hereford-Angus cows, naturally endemic for anaplasmosis, was used in this study. Animals were grazed in five different pastures: Goheen (G), Texas Hog (TH), Shane Creek (SC), South Konza (SK), and North Konza (NK) located in the Konza Prairie research facility (39.1069° N, 96.6091° W) (May–Oct) 5 miles south of Manhattan, KS, USA. Cow age ranged from 1 to 16 years old. Pastures were stocked at a rate of 3.24 hectares per cow for 150 days. Pastures were dominated by big bluestem (*Andropogon gerardii*), little bluestem (*Schizachyrium scoparium*), Indian grass (*Panicum virgatum*), and sideoats grama (*Boutelua curtipendula*) [27].

#### 2.1.2. Treatment Groups

Treatment groups were randomly assigned by pasture allocation. Pasture location and allocation of animals into their respective pastures were determined based on their involvement in a concurrent unrelated study. Therefore, animal movement amongst the pastures to correct for an unequal number of cows per treatment group was prohibited. Chlortetracycline-medicated mineral formulations (700, 5000, 6000, 8000 g CTC/ton) were randomly assigned to pastures TH, SC, SK, and NK, respectively (i.e., 700 g CTC/ton Aureo Anaplaz C700 Pressed [Sweetlix Livestock Supplements, Mankato, MN, USA]; 5000 g CTC/ton, Purina Anaplasmosis Block [Purina Animal Nutrition, Gray Summit, MO, USA]; 6000 g CTC/ton, Stockmaster Aureo FC C6000 Mineral [Hubbard Feeds, Mankato, MN, USA]; 8000 g CTC/ton, MoorMan’s Special Range Minerals AU 168XFE [ADM Animal Nutrition, Quincy, IL, USA]; Table 1). Pasture G served as the untreated control, receiving a non-medicated free-choice mineral supplement (i.e., 0 g CTC/ton). The remaining herds, in their respective pastures, were provided one of the four approved free-choice CTC-medicated mineral formulations ad libitum from early June through late October in 2017. The animals in the study had not been fed CTC for at least 17 months prior to this study; undetectable plasma-CTC concentrations were confirmed in cows prior to the initiation of this study. Details of CTC-medicated mineral administration to these cattle herds have previously been published [27]. Briefly, cattle were acclimated to non-medicated mineral for 14 days prior to sample collection. Mineral feeders were monitored and filled once a week. One mineral feeder was provided for every 10 cows in individual pastures.

### 2.2. Sample Collection & Processing

#### 2.2.1. Fecal Collection

Study animals were gathered from their grazing pasture and fecal samples were collected approximately monthly from June 2017 to October 2017. Data were collected at six time points, including 6/5/17 (Baseline), 6/26–6/28 (JUN), 7/27/17 (JUL), 8/30/17 (AUG), 9/25/17 (SEP), and 10/23–10/31 (OCT). At Baseline, fecal samples were collected from six (*n* = 6) or ten (*n* = 10) randomly selected cattle per treatment group (Supplemental Table S1). The same subset of cattle were utilized at each time for subsequent fecal collections and blood draw. Some time points did not have all ten fecal samples collected because the cattle could not be feasibly gathered and restrained. After collection, aliquots of individual fecal samples were resuspended in glycerol in two 10 mL tubes at an approximate 1:1 ratio of the fecal matter: glycerol and stored at −80 °C.

#### 2.2.2. *Escherichia coli* Isolation

Fecal samples were cultured for *E. coli* isolates using our previously published procedures [28,29]. In brief, approximately 1 g of fecal sample was homogenized with 10 mL of phosphate-buffered saline. Then, 50 µL of the fecal suspension was plated onto a MacConkey Agar and incubated at 37 °C for 24 h. For each fecal sample, two putative lactose-fermenting isolates (i.e., biological replicates) were selected from each plate for further characterization. Each isolate was individually streaked onto a blood agar plate (Remel, Lenexa, KS, USA) and incubated at 37 °C for 24 h. The isolates were confirmed as *E. coli* via the Spot Indole Test (described below). For each fecal sample, two *E. coli* isolates were identified and tested for CTC susceptibility (described below).

#### 2.2.3. Spot Indole Test

Cultured blood agar plates were removed from the incubator after 24 h. Filter paper placed in a petri dish was saturated with James’ reagent (bioMérieux, St. Louis, MO, USA). A bacteriologic loop was used to remove a small portion of the bacterial isolate from the agar surface and the sample was rubbed onto the filter paper. A bacterial isolate is positive for indole if the filter paper turns a pink color within 30 s (*E. coli* positive). No color development is considered negative for indole production (*E. coli* negative). All confirmed indole positive *E. coli* isolates were stored in cryoprotectant beads CryoBeads™ (KEY Scientific Products, Stanford, TX, USA) at −80 °C. Two *E. coli* isolates were stored for each sample.

#### 2.2.4. Chlortetracycline (CTC) Susceptibility Testing of *Escherichia coli* Isolates 

The micro-broth dilution method was used to determine the minimal inhibitory concentrations (MIC) of *E. coli* isolates in response to CTC. The *E. coli* isolates were tested as previously described [30,31] using a modified version of the CLSI guidelines [32]. The modification was using a slightly different two-fold dilution scheme (100, 50, 25, 12.5, 6.25, 3.125, 1.56, 0.78, 0.39, and 0.195 µg/mL) instead of (128, 64, 32, 16, 8, 4, 2, 1, 0.5, and 0.25 µg/mL). Each isolate stored in cryoprotectant beads was streaked onto a blood agar plate and incubated at 37 °C for 24 h. Individual colonies were suspended in 10 mL Mueller-Hinton II (MH) Broth and the turbidity was adjusted to 0.5 McFarland turbidity standards. A 1:100 dilution of culture was prepared by adding 50 µL (0.05 mL) of the isolate culture into 5 mL of MH broth. 

Next, a micro-dilution was prepared in a 96-well U-bottom plate. The first column of the plate served as a bacterial growth control (no antibiotic), while the second column served as antibiotic control (no bacterial inoculum). Forty microliters of the CTC stock solution (100 µg/mL) was added to the plate beginning in column #2. This was mixed well by repeat pipetting and a serial dilution was achieved by mixing and transferring 100 µL from column #2 to column #3 and so on until the last column. The remaining 100 µL from the last column was then discarded. Each column except column #2 were inoculated with 100 µL of dilute culture. Lastly, 100 µL of MH broth was added to column #2 (antibiotic control). The final concentrations of CTC in each well are as follows: 100, 50, 25, 12.5, 6.25, 3.125, 1.56, 0.78, 0.39, and 0.195 µg/mL. Plates were incubated at 37 °C for 18 to 24 h.

The MIC for each *E. coli* isolate was reported as the lowest concentration of the antimicrobial that inhibited visible bacterial growth. For this study, MIC refers to the geometric mean of MICs for two isolates derived from the same sample. For *E. coli*, isolates are classified as susceptible (≤4 μg/mL), intermediate (8 μg/mL), or resistant (≥16 μg/mL) to CTC, based on guidelines established by the CLSI [32]. 

#### 2.2.5. CTC Quantification 

Blood was drawn from each animal at the same five, approximately monthly, post-treatment initiation time points when fecal samples were collected. At each time point, approximately 6 mL of blood was collected via coccygeal venipuncture directly into a lithium heparin tube from each cow (Vacuette, Greiner Bio-One North America Inc., Monroe, NC, USA). Blood samples were spun down, plasma collected, and stored at −80 °C until plasma-CTC analysis. Plasma-CTC concentrations for this sample set were previously reported in Reppert et al. (2020) [27]. 

### 2.3. Statistical Analysis

For the purpose of statistical modeling, MIC of fecal isolates that fell above the upper limit of detection (50 μg/mL) were replaced by twice the detection limit (100 μg/mL); blood plasma CTC concentration levels that fell below the lower limit of detection (1 ng/mL) were replaced by half of the detection limit (0.5 ng/mL).

The data were subjected to natural-log transformation before linear mixed model analysis to better achieve the model assumptions.

The linear mixed model for log-transformed MIC contained the fixed effects of treatment group (0 g CTC/ton (G), 700 g CTC/ton (TH), 5000 g CTC/ton (SC), 6000 g CTC/ton (SK) 8000 g CTC/ton (NK)), time point (Baseline, JUN, JUL, AUG, SEPT, OCT) and their interaction. Random effects of the model included animal ID (the error term vector corresponding to repeated measurement over time) and animal-ID-by-time-point (the error term vector corresponding to subsampling of two isolates). The variance-covariance structure of animal ID was taken as compound symmetry according to the model fitting criteria. The variance-covariance structure of animal-ID-by-time-point was taken as variance components. The age of cattle served as a covariate. Baseline MIC did not serve as the covariate because it was not available for four cattle. Instead, the present statistical approach considered Baseline as one of the time point levels and adjusted for the Baseline effect via customizing the linear functions of model fixed effects during hypotheses testing and parameter estimation. 

The linear mixed model for log-transformed CTC plasma concentration contained the fixed effects of treatment group, time point (JUN, JUL, AUG, SEPT, OCT), and their interaction. Random effects of the model included animal ID (error term vector corresponding to repeated measurement over time). The variance-covariance structure of animal ID was taken as first-order autoregressive according to the model fitting criteria. The age of cattle served as a covariate. 

The treatment effect was assessed via back-transforming least squares means (corresponding to medians on the original scale), their standard error (SE), and mean differences (corresponding to ratios of medians on the original scale). Pairwise comparisons were carried out using the 2-sided test. Disregarding results of the test for treatment-by-time point interaction, treatment groups were compared within each time point (Table 2); time points were compared within each treatment group; main effect of treatment was also reported and compared (Table 3). No multiplicity adjustment was applied. SAS Statistical analysis was executed via Statistical Analysis Software (SAS version 9.4; Cary, NC, USA) PROC MIXED with option DDFM = KR. 

For the purposes of calculating the MIC_50_ and MIC_90_, the test population consisted of animals across all study groups provided CTC-medicated feed products, with a CTC measurement from at least two of the five sampling time points and a MIC value measured at Baseline or final time point (May: 32 animals, October: 25 animals). The MIC_50_ represents the MIC value at which at least 50% of the isolates in a test population are inhibited; it is equivalent to the median MIC value among isolates sourced from the same population. The MIC_90_ represents the MIC value at which at least 90% of the isolates within a test population are inhibited; the 90th percentile.

## 3. Results

### 3.1. Experimental Group Demographics and Sampling

Study animals consisted of cows and heifers from a natural anaplasmosis-endemic cow-calf herd routinely maintained on pasture from May to October. Cattle ranged in age from 1 to 16, however, the animals in the untreated control group (G) were all heifers (Table 1). While there was considerable age variability within treatment groups and between the treatment and control groups, there was no statistical difference between age and CTC-medicated mineral formulation or MIC values. Fecal samples were collected from six (*n* = 6) or ten (*n* = 10) randomly selected cows per treatment group (Supplemental Table S1). The same cattle were utilized each time for subsequent fecal collections and blood draw. Some time points did not have all ten (or six for group NK) fecal samples collected because the cattle could not be feasibly gathered and restrained on pasture (Supplemental Table S1).

### 3.2. Evaluation of Changes in E. coli Susceptibility in Cattle Offered Different Free-Choice CTC-Medicated Feed Products

Free-choice administration of CTC is frequently used to control anaplasmosis in grazing cattle; however, long-term antimicrobial use may promote the development of AMR in off-target microbial species such as *E. coli* in the bovine gastrointestinal tract. To evaluate the effect of extended CTC administration for anaplasmosis control on off-target microbes, CTC susceptibility was evaluated in *E. coli* isolated from cattle herds provided different FDA-approved, free-choice CTC-medicated feed formulations for five consecutive months. Specifically, the susceptibility of *E. coli* isolates to CTC was evaluated by determining the MIC monthly over the course of CTC administration. The median, minimum, and maximum MIC values for all study groups at each sampling time point are provided in Supplemental Table S2. Overall, *E. coli* isolates from the NK group (8000 g CTC/ton) had the highest median MIC at four of the six time points, with five of the median MIC values falling into the CLSI resistant (≥16 μg/mL) category. In contrast, *E. coli* isolates from the G group (0 g CTC/ton) demonstrated the most susceptibility to CTC, with the median MIC at two, four, and one time point(s) falling into the CLSI susceptible (<4 μg/mL), intermediate (8 μg/mL), and resistant category (≥16 μg/mL), respectively.

**Figure 1:**
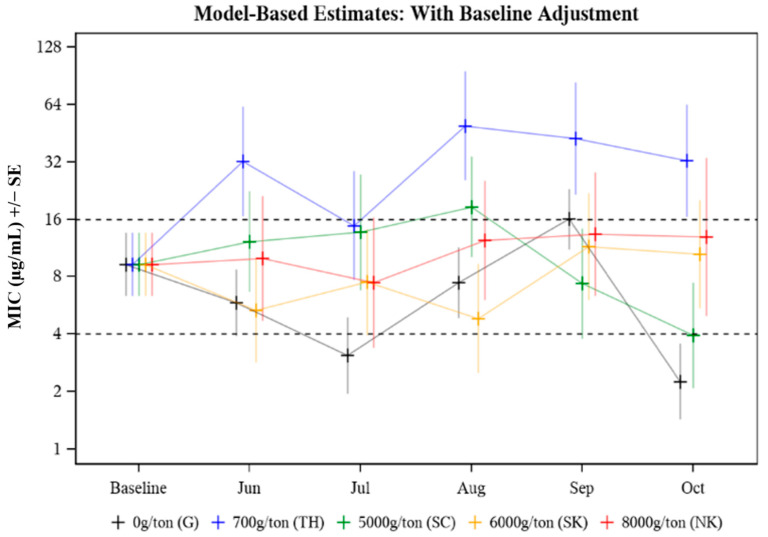
Model-based estimates of median minimum inhibitory concentration (MIC) for *E. coli* isolated from cattle herds provided different FDA-approved CTC-medicated mineral formulations to control *A. marginale.* Baseline adjusted MIC for *E. coli* recovered from cattle treated with different CTC-medicated mineral formulations for six months.

The MIC was evaluated in response to the following effects: animal age (*p* = 0.578), treatment group (*p* = 0.037), time (*p* = 0.090), and treatment group by time point (*p* = 0.075). The only significant effect was treatment group (*p* = 0.037) (Table 2) where an overall significant difference between median treatment group MICs was observed in August (*p* = 0.027) and October (*p* = 0.009) (Table 3). In August, the median MIC in the 700 g/ton pasture was 10.20 times as high as that in the 6000 g/ton pasture (*p* = 0.002) (Table 3). In October, the median MIC value in the 700 g CTC/ton pasture was 8.21 times as high as that in the 5000 g CTC/ton pasture (*p* = 0.006) (Table 3). The median MIC data for *E. coli* isolated from cattle treated with different CTC-medicated mineral formulations over time (Baseline to October) is presented in Figure 1. Baseline adjustments were made so each treatment and control group had the same starting MIC value for comparison purposes, with Baseline median MIC values falling in the intermediate (8 μg/mL) CLSI category. Median MIC remained in the intermediate CLSI category throughout the study period with a few exceptions where transient differences were observed (Figure 1, Table 3). Study group TH (700 g CTC/ton) cows had consistently higher median MICs compared to the other groups (Figure 1). While the variability of MIC values was evident across all treatment groups and sampling time points, no distinct pattern (i.e., the trend towards greater resistance as treatment continued) was observed. 

Collectively, from study groups provided a CTC-medicated feed product (TH, SC, SK, NK), the distribution of *E. coli* isolate CTC MIC values prior to initiation of CTC treatment and after five months of treatment are presented in Supplemental Figure S1. Pre-treatment MIC levels (Baseline) ranged from 0–50 µg/mL with approximately half of the animals with a MIC between 6.25–50 µg/mL (intermediate to resistant CLSI category) and approximately 15% with a MIC value > 25 µg/mL (Supplemental Figure S1). Post-treatment MIC levels (October) ranged from 1.56–50 µg/mL but were more consolidated with no *E. coli* isolates observed to have a MIC < 1.56 µg/mL (compared to approximately 13% at Baseline) and less than 5% of isolates with MIC values above 25 µg/mL (compared to approximately 15% at Baseline) (Supplemental Figure S1). Even though *E. coli* isolates with extremely high and low MIC values were less prevalent in October, overall *E. coli* isolate MIC values had a uniform distribution at both Baseline (May) and the final time point (October) with isolates falling within susceptible to resistant CLSI guideline categories.

The MIC_50_ and MIC_90_ were calculated from the cohort of animals used in this study from each treatment group provided a CTC-medicated feed product (TH, SC, SK, NK). Among study animals, their cumulative Baseline MIC_50_ was 10.7 µg/mL and their cumulative MIC_90_ was 50 µg/mL. After being provided a CTC-medicated feed product, their cumulative MIC_50_ and MIC_90_ remained similar at 12.5 µg/mL and decreased to 25 µg/mL, respectively. 

### 3.3. Association/Relationship between MIC and Plasma CTC Levels

In a free-choice administration system, individual animals balance their own diet and choose whether and how much to feed on provided medicated product. Therefore, some animals may consume more or less medicated product on a more or less frequent basis while other animals may consume none. The daily amount of CTC-medicated feed product consumed by individual animals in each study group was not calculated as these cattle were maintained on pasture throughout the study period. Because of expected heterogeneity in CTC-medicated mineral consumption and the half-life of CTC, *E. coli* isolate MIC values were compared among study animals based on the frequency of having detectable plasma CTC from matched blood samples. 

The raw plasma CTC concentration data, previously published in Reppert et al., 2020 [27], was re-evaluated to investigate the association between plasma CTC level and changes in MIC collectively among animals provided a CTC-medicated feed product (TH, SC, SK, NK). Plasma CTC concentration data was evaluated in relation to age, treatment group, time, and treatment effect by time point. The only significant effect of plasma CTC concentration was the treatment effect by time point (*p* = value 0.036). Within time point, there was a significant difference in plasma CTC concentration among treatment groups at two of the five time points: June (*p* = 0.0011) and October (*p* = 0.0389) (Supplemental Figure S2). The median herd-wide plasma CTC concentrations, among all cattle with detectable plasma CTC, ranged between 3.9 to 45.3 ng/mL over the treatment period. Of the cohort of cattle evaluated in the present study (*n* = 36 total from groups TH, SC, SK, NK), plasma CTC was detectable in 137 out of 180 possible samples (36 animals × 4 CTC-treatment groups × 5 time points). Despite the expected intake variability between animals, plasma CTC concentrations remained similar throughout the study.

**Figure 2:**
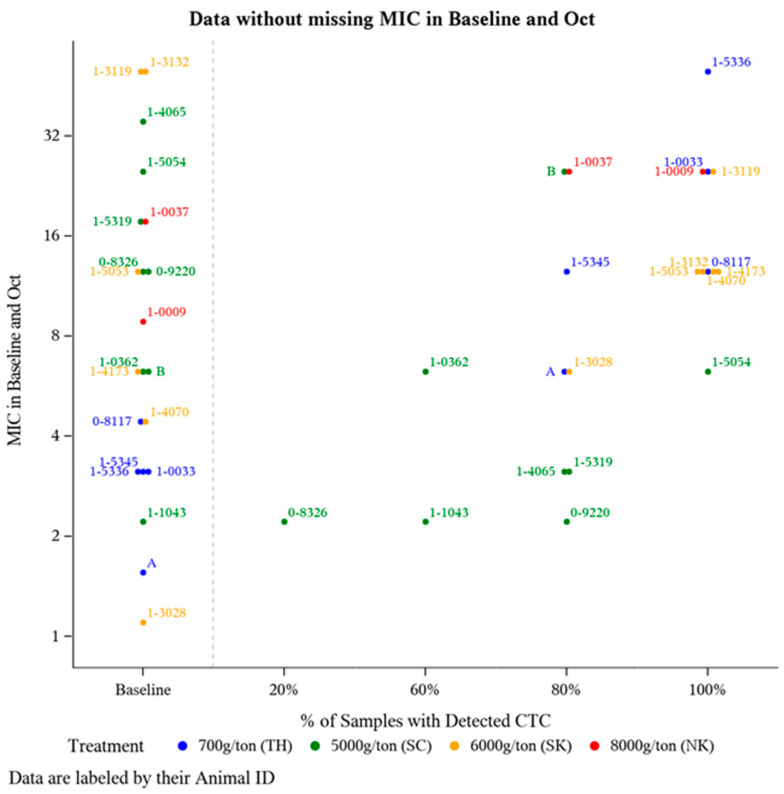
Distribution of *E. coli* isolate MIC levels pre-CTC treatment versus post-CTC treatment relative to the frequency of detectable plasma CTC levels. Of the 36 animals with detectable plasma CTC concentration at a minimum of 2 sampling time points, 21 animals had a MIC measurement at both Baseline (May) and the final time point in October.

To further investigate potential changes in *E. coli* CTC susceptibility as a result of offering CTC for a prolonged period of time, *E. coli* isolate MIC was compared from a subset of animals (*n* = 21) that had detectable plasma CTC concentrations during at least two time points (suggests a greater likelihood for routine CTC-medicated mineral consumption). Frequency of detectable plasma CTC is the number of sampling time points an individual animal had a detectable amount (>1 ng/mL) of CTC in their plasma out of the five post-treatment sampling time points (June-October). For these 21 animals, *E. coli* isolate MIC was compared at Baseline and after five months of continuous CTC provision (October). The majority of these 21 animals harbored *E. coli* isolates with some degree of resistance prior to CTC being administered (Figure 2), with 71% (15/21) having Baseline MIC value >4 µg/mL (intermediate CLSI category) (Figure 2). Though not statistically analyzed, a positive association between the frequency of detecting plasma CTC and *E. coli* isolate MIC was observed (Figure 2). 

## 4. Discussion

Antimicrobial use in food-producing animals is a critical and unavoidable practice for the treatment of diseases in which vaccines or alternative therapies are not available. Antimicrobial resistance is a by-product that needs to be carefully considered when treating a large number of animals for an extended period of time, as it threatens the effective lifespan of these drugs to treat both animal and human diseases. This study investigated changes in CTC susceptibly of *E. coli* isolated from pastured cattle provided different free-choice CTC-medicated mineral formulations for the control of anaplasmosis. Anaplasmosis occurs in temperate, tropical, and subtropical regions worldwide and has been reported in almost every state in the U.S. [9]. Pastured cattle frequently receive continuous CTC-medicated feed products for extended periods of time to control anaplasmosis; however, the impact of this antimicrobial usage on other microbial species carried by cattle, such as *E. coli*, is unknown. 

There are currently four FDA-approved free-choice CTC-medicated mineral formulations (700, 5000, 6000, 8000 g/ton) labeled for “the control of active anaplasmosis”. Regardless of formulation, all are intended to deliver in the target dose range of 0.5 to 2.0 mg CTC per lb of body weight per animal per day. Free-choice medicated mineral is a common method of antimicrobial-based anaplasmosis control used for pastured cattle. Feeding free-choice mineral assumes each individual animal uses its natural instinctive ability to self-regulate intake of the medicated product to satisfy nutrient requirements. With no limit on the duration of use, approved CTC-medicated feed products may be fed for an extended period of time (months to all year), as long as the producer has a valid VFD. In this study, we addressed: (1) the impact of CTC-medicated mineral formulation on the susceptibility profile of *E. coli*; and (2) how the duration of exposure to the mineral product may facilitate the expansion of resistant *E. coli*.

The median *E. coli* isolate MIC values were compared among study groups of pastured cattle provided no CTC (pasture G, untreated control) or one of four different formulations of CTC-medicated mineral from prior to CTC exposure through five consecutive months of CTC exposure (Table 3). Changes in MIC over the treatment period (Baseline-Oct) were evaluated by Baseline adjusting all MIC values to fall in the intermediate CLSI category for *E. coli* isolated from individual fecal samples collected pre-treatment (Figure 1). The MIC in response to treatment (*p* = 0.037) was statistically significant; specifically driven by differences in the median MICs among the five study groups in August (*p* = 0.027) and October (*p* = 0.009) (Table 3). The plotted MIC values only represent a subset of the population (*n* = 6 or *n* = 10) from each pasture; therefore, higher MIC values may be a result of fewer data points skewing the data. While the variability of MIC values was evident across all treatment groups and sampling time points, no distinct pattern (i.e., the trend towards greater resistance as treatment continued) was observed. Despite some differences between individual groups at specific time points, most evaluated *E. coli* isolates remained in the intermediate (8 μg/mL) CLSI category (Figure 1). 

The physical form of CTC-medicated feed products varied between treatment groups. Pastures TH and SC that had access to 700 g/ton and 5000 g/ton, respectively, received supplemental CTC-medicated mineral in block form, while pastures SK and NK that had access to 6000 g/ton and 8000 g/ton, respectively, received supplemental CTC-medicated mineral in granular form. The block-style supplements are formulated with molasses products to facilitate increased palatability. Therefore, animals in the 700 g/ton pasture may have consumed more CTC, subsequently affecting the MIC values of the animals in that pasture. We can theorize, block-style supplements formulated with molasses may have encouraged greater or more regular consumption, which could drive *E. coli* resistance to CTC. Similar to our findings, Reppert et al., 2020 [27] found that cows exposed to 700 g/ton CTC medicated mineral block had significantly higher dose-adjusted plasma CTC concentrations compared with other pasture groups (*p* < 0.0001). This linear relationship of plasma CTC level and MIC could indicate that exposure to CTC increases the likelihood of subsequent CTC resistance.

The cumulative effect of exposure to CTC and subsequent *E. coli* susceptibility over the treatment period was evaluated by comparing *E. coli* isolates derived from animals prior to CTC treatment (Baseline) compared to animals that had access to CTC for the previous five months (October) (Supplemental Figure S1). As expected, we saw a slight increase in *E. coli* resistance to CTC over the treatment period (Supplemental Figure S1). A previous study addressing the concern of tetracycline-resistant *E. coli* following 5-days of in-feed CTC administration found a temporary increase in fecal tetracycline-resistant *E. coli* [19]. This is consistent with our findings, where approximately 12% of the isolates from the Baseline initially in the susceptible category (0, 1.56 µg/mL) were absent during the final sampling time point, suggesting reduced population susceptibility to CTC. However, this increase in resistance is small and may be attributed to the limited sample size (May: 32 animals, October: 25 animals). In addition to sample size, the two isolates used for testing may not provide a clear representation of the entire *E. coli* bacterial population in the bovine fecal microbiome.

For all treatment groups (TH, SC, SK, NK), at Baseline the MIC_50_ was 10.7 µg/mL and the MIC_90_ was 50 µg/mL. Similarly, for all treatment groups, the final time point in October the MIC_50_ was 12.5 µg/mL and the MIC_90_ was 25 µg/mL. Although all animals in each treatment group had access to one of the medicated mineral formulations, this does not guarantee each animal regularly consumed a consistent amount of CTC-medicated product (discussed below). Furthermore, MIC_50_ and MIC_90_ are metrics commonly used in antimicrobial susceptibility analyses for large populations; however, due to our limited sample size (May: 32 animals, October: 25 animals), the value of these metrics decreases. 

Dose-adjusted plasma CTC concentrations were previously reported in Reppert et al., 2020 [27]. The raw plasma CTC concentration data was re-evaluated in this study to investigate the association frequency of detectable CTC level and *E. coli* isolate MIC. A range of variability in plasma CTC concentrations was expected, since the CTC-medicated mineral product is provided in a free-choice manner, allowing individual animals to consume varying amounts based on individual needs. However, all CTC-medicated mineral formulations are intended to deliver the same dosage (0.5 to 2.0 mg CTC/lb of BW daily) of CTC. Despite variable intake expected in a pasture setting, CTC concentrations remained overall similar between treatment groups (Supplemental Figure S2). The mean plasma CTC concentrations determined in this study are consistent with a previous study where cattle were administered hand-fed CTC-medicated product on pasture. The mean plasma CTC concentration in cattle on pasture ranged from <0.1–51.1 ng/mL [33]. As samples (fecal and blood) were collected only once per month; individual animal mineral consumption patterns, including the timing of last medicated mineral consumption prior to sample collection, may impact fecal *E. coli* isolate MIC and blood plasma CTC level. In steers that were hand-fed CTC in a feedlot setting, the mean half-life of CTC was determined to be 16.2 h [34]. Using this half-life, in an animal that consumed CTC-medicated mineral 24 h prior to sampling collection, the plasma CTC concentration would be near elimination. Universal and uniform consumption of free-choice mineral produced by individuals in a herd should not be assumed [35]. 

While each treatment group was provided one of the FDA-approved CTC-medicated mineral formulations (all intended to deliver 0.5 to 2.0 mg CTC/lb of BW daily), this does not ensure all the study animals in each treatment pasture equally consumed the medicated mineral. Variation in mineral intake can be attributed to environmental conditions, pasture topography and size, soil fertility, climate changes, location of water and shade, access/location of mineral feeders, frequency of sampling, and more. To address this likelihood of variable medicated mineral intake and its impact on *E. coli* isolate MIC values, a subset of animals (*n* = 36) were evaluated based on the percentage of sampling time points with where they had detectable CTC levels (Figure 2). Unsurprisingly, most animals at Baseline (prior to any exposure to CTC), harbored *E. coli* isolates with CTC MIC values >4 µg/mL (Figure 2). According to CLSI guidelines, this breakpoint indicates the majority of the isolates at Baseline were in either the intermediate or resistance categories. Tetracycline resistance among *E. coli* in cattle is relatively common [23]. This is consistent with our findings displayed at Baseline in Figure 2, there is a level of inherent resistance within the microbial community independent of CTC exposure. These findings support previous research showing that resistance to tetracycline is widespread [36] and prevalent in the ruminant intestinal microflora even when animals are not fed antibiotics [25]. Additionally, an upward trend in (MIC) resistance relative to the frequency of detectable CTC was observed in the *E. coli* isolates (Figure 2). 

Measuring drug intake and evaluating drug efficacy based on intake in pastured cattle provided free-choice medicated feed can be challenging due to sampling access as well as a multitude of biotic and abiotic variables that can influence consumption of the medicated product [37]. Most commonly, producers will have their herd in a pasture with access to *ad libitum* forage and supply a free-choice mineral supplement, as was done in this study. The inherent variability of CTC intake makes it challenging to explore the linkage between CTC consumption and antimicrobial resistance in *E. coli*. Future studies are needed to evaluate the off-target impact of long-term antimicrobial-based anaplasmosis, control on other microbial species, in different production settings, among cattle of various signalments, with greater sample sizes, and more sampling time points. 

## 5. Conclusions

The susceptibly profile of *E. coli* isolates was investigated in anaplasmosis-endemic pastured cattle herds provided different free-choice formulations of CTC-medicated mineral for five consecutive months to control anaplasmosis. No pattern or clear development of resistance was observed in *E. coli* isolated from CTC-treated cattle; however, variations in medicated mineral consumption patterns among sampled cattle may have affected the results.

While the limitations discussed above hinder our ability to state definitive conclusions, the aspects of the study align with real-world scenarios. Antimicrobial-based anaplasmosis treatment and control options are limited and continuously becoming more regulated. To preserve the efficacy and longevity of medically-important antimicrobials, it is critical to periodically (re)evaluate their effectiveness against their intended targets (i.e., *A. marginale*) as well as investigate potential off-target implications (i.e., promoting antimicrobial resistance in off-target *E. coli*). Additional studies evaluating the off-target impact of long-term antimicrobial-based anaplasmosis control in different production settings, with greater sample sizes and sampling time points are needed. The relationship between continuous CTC administration and development of antimicrobial resistance of off-target species is complicated and requires continued investigation.

## Figures and Tables

**Table 1:** Treatment groups and the number of fecal samples collected for analysis.

Treatment Group (CTC) ^1^	Control-or-Medicated Feed	Pasture	Max Number of Animals Sampled (Fecal Samples) ^2^ per Time Point	Total Number of Fecal Samples Tested over the Study Period per Group
0 g/ton (G)	Control	Goheen	10	51
700 g/ton (TH)	700 g/ton	Texas Hog	10	50
5000 g/ton (SC)	5000 g/ton	Shane Creek	10	56
6000 g/ton (SK)	6000 g/ton	South Konza	10	59
8000 g/ton (NK)	8000 g/ton	North Konza	6	29

^1^ FDA-approved free-choice CTC-medicated feed formulations. All are intended to deliver 0.5 to 2.0 mg CTC/lb body weight/day. ^2^ Fecal samples were collected from a subset of randomly selected cattle per treatment group. The same cattle were utilized each time for subsequent fecal collections.

**Table 2:** Across-time comparison of median MIC values in treatment groups of pastured cattle provided four different FDA-approved formulations of CTC-medicated mineral products. Treatment groups with the same letter were not significantly different in their pairwise comparison.

*p*-Value for Testing Overall Treatment Effect	Treatment	MIC (ug/mL) Baseline Adj. Median +/− SE		Ratio (*p*-Value) Comparison Between Groups			
				700 g/ton (T)	5000 g/ton (SC)	6000 g/ton (SK)	8000 g/ton (NK)
0.037	0 g/ton (G)	5.5 +/− 1.2	A	0.17 (0.003)	0.56 (0.290)	0.73 (0.585)	0.50 (0.276)
	700 g/ton (TH)	31.8 +/− 18.0	B	--	3.24 (0.038)	4.25 (0.014)	2.89 (0.104)
	5000 g/ton (SC)	9.8 +/− 5.3	A	--	--	1.31 (0.630)	0.89 (0.856)
	6000 g/ton (SK)	7.5 +/− 4.2	A	--	--	--	0.68 (0.553)
	8000 g/ton (NK)	11.0 +/− 6.9	AB	--	--	--	--

**Table 3:** Within-time comparison of median MIC values in treatment groups of pastured cattle provided four different FDA-approved formulations of CTC-medicated mineral throughout five months of continuous CTC treatment. Treatment groups with the same letter were not significantly different in their pairwise comparison.

Time	*p*-Value for Testing Overall Treatment Effect	Treatment	MIC (μg/mL) Baseline-Adj. Median +/− SE		Ratio (*p*-Value) Comparison Between Groups			
					700 g/ton (TH)	5000 g/ton (SC)	6000 g/ton (SK)	8000 g/ton (NK)
Baseline	--	--	9.3 +/− 3.5		--	--	--	--
Jun	0.113	0 g/ton (G)	5.9 +/− 2.4	A	0.18 (0.023)	0.48 (0.295)	1.10 (0.899)	0.59 (0.519)
		700 g/ton (TH)	32.3 +/− 21.3	B	--	2.63 (0.177)	6.03 (0.016)	3.22 (0.163)
		5000 g/ton (SC)	12.3 +/− 7.5	AB	--	--	2.29 (0.233)	1.22 (0.801)
		6000 g/ton (SK)	5.3 +/− 3.4	A	--	--	--	0.53 (0.445)
		8000 g/ton (NK)	10.0 +/− 7.5	AB	--	--	--	--
Jul	0.287	0 g/ton (G)	3.1 +/− 1.4	A	0.21 (0.045)	0.23 (0.069)	0.41 (0.250)	0.41 (0.324)
		700 g/ton (TH)	14.8 +/− 9.8	B	--	1.08 (0.919)	1.98 (0.360)	1.99 (0.431)
		5000 g/ton (SC)	13.7 +/− 9.6	AB	--	--	1.83 (0.443)	1.83 (0.504)
		6000 g/ton (SK)	7.5 +/− 4.8	AB	--	--	--	1.00 (0.996)
		8000 g/ton (NK)	7.5 +/− 5.9	AB	--	--	--	--
Aug	0.027	0 g/ton (G)	7.5 +/− 3.2	A	0.15 (0.014)	0.40 (0.206)	1.54 (0.570)	0.60 (0.535)
		700 g/ton (TH)	49.4 +/− 32.4	B	--	2.66 (0.173)	10.20 (0.002)	3.98 (0.091)
		5000 g/ton (SC)	18.6 +/− 11.3	AB	--	--	3.84 (0.062)	1.50 (0.603)
		6000 g/ton (SK)	4.8 +/− 3.2	A	--	--	--	0.39 (0.250)
		8000 g/ton (NK)	12.4 +/− 8.9	AB	--	--	--	--
Sep	0.251	0 g/ton (G)	16.0 +/− 5.8	AB	0.38 (0.190)	2.18 (0.294)	1.39 (0.644)	1.20 (0.823)
		700 g/ton (TH)	42.4 +/− 28.7	B	--	5.75 (0.027)	3.68 (0.088)	3.17 (0.177)
		5000 g/ton (SC)	7.4 +/− 4.9	A	--	--	0.64 (0.556)	0.55 (0.484)
		6000 g/ton (SK)	11.5 +/− 7.4	A	--	--	--	0.86 (0.857)
		8000 g/ton (NK)	13.4 +/− 10.0	AB	--	--	--	--
Oct	0.009	0 g/ton (G)	2.3 +/− 1.0	A	0.07 (<0.001)	0.57 (0.462)	0.22 (0.050)	0.17 (0.096)
		700 g/ton (TH)	32.5 +/− 21.9	C	--	8.21 (0.006)	3.09 (0.145)	2.51 (0.378)
		5000 g/ton (SC)	4.0 +/− 2.5	AB	--	--	0.38 (0.186)	0.31 (0.244)
		6000 g/ton (SK)	10.5 +/− 6.9	BC	--	--	--	0.81 (0.839)
		8000 g/ton (NK)	12.9 +/− 12.4	AB	--	--	--	--

## Data Availability

All data generated or analyzed during this study are included in the published article or Supplementary files.

## Supplementary Materials

The following are available online at https://www.mdpi.com/article/10.3390/microorganisms9122495/s1, Table S1: # of animals fecal sampled within each treatment group at each time point, Table S2: Median, minimum, and maximum MIC values for all study groups at each sampling time point, Figure S1: Distribution of MIC values from *E. coli* isolates derived from study groups provided a CTC-medicated feed product at Baseline (prior to CTC exposure) and October (5 months continuous treatment). This data was derived from a subset of the animals that had a MIC value available at baseline or the final sampling time point (Baseline: 32 animals, October: 25 animals). CLSI breakpoints for *E. coli* susceptible (≤4 μg/mL), intermediate (8 μg/mL), or resistant (≥16 μg/mL) to CTC, Figure S2: Model prediction of mean plasma CTC values in each treatment group over the treatment period. Determined plasma CTC values in cattle from each treatment group.
