# Supplementary material for: Effect of Protracted Free-Choice Chlortetracycline-Medicated Mineral for Anaplasmosis Control on Escherichia coli Chlortetracycline Resistance Profile from Pastured Beef Cattle
Source: Microorganisms. 2021 Dec 2;9(12):2495. doi: 10.3390/microorganisms9122495 (PMC8704331; doi:10.3390/microorganisms9122495)
Supplement: Supplementary file 1 [file microorganisms-09-02495-s001.zip › microorganisms-1468391-supplementary.pdf]

Supplemental Table S1. # of animals fecal sampled within each treatment group at each time point.

Pasture	Treatment group[1]	Number of animals fecal sampled at each time point						Total number of fecal samples tested per pasture
		Baseline[2]	June	July	Aug	Sept	Oct	
Goheen	0 g/ton	9	9	8	8	10	7	51
Texas Hog	700 g/ton	10	9	8	8	7	8	50
Shane Creek	5,000 g/ton	10	10	9	10	9	8	56
South Konza	6,000 g/ton	10	10	10	10	10	9	59
North Konza	8,000 g/ton	6	6	4	6	5	2	29
								245

[1] All formulations are intended to deliver 0.5 to 2.0 mg CTC/lb body weight/day.
[2] Baseline is prior to any exposure to CTC.

Supplemental Table S2. Median, minimum, and maximum MIC values for all study groups at each sampling time point.

		MIC: Geometric Mean of 2 Isolates				
		**Number of Animals**	**Median**	**Min**	**Max**	**Number of Isolates >50**
**Baseline**	**0g/ton (G)**	9	8.839	0.552	50	0
	**700g/ton (TH)**	8	3.125	1.56	50	0
	**5000g/ton (SC)**	10	12.5	1.56	35.36	1
	**6000g/ton (SK)**	8	9.375	1.103	50	0
	**8000g/ton (NK)**	6	17.68	8.839	50	0
**Jun**	**0g/ton (G)**	8	6.247	1.56	35.36	0
	**700g/ton (TH)**	8	21.34	0.78	>50	2
	**5000g/ton (SC)**	10	18.75	2.208	50	0
	**6000g/ton (SK)**	10	6.247	0.39	25	0
	**8000g/ton (NK)**	5	25	12.5	35.36	0
**Jul**	**0g/ton (G)**	6	3.125	1.56	6.25	0
	**700g/ton (TH)**	8	6.627	0.552	50	0
	**5000g/ton (SC)**	5	17.68	2.208	50	0
	**6000g/ton (SK)**	9	6.25	0.78	50	0
	**8000g/ton (NK)**	4	12.5	12.5	25	0
**Aug**	**0g/ton (G)**	7	6.25	1.56	25	0
	**700g/ton (TH)**	8	21.34	6.25	50	0
	**5000g/ton (SC)**	10	25	4.419	50	0
	**6000g/ton (SK)**	8	5.333	1.56	50	1
	**8000g/ton (NK)**	6	25	12.5	50	0
**Sep**	**0g/ton (G)**	10	26.52	0.78	>50	2
	**700g/ton (TH)**	7	25	3.125	50	0
	**5000g/ton (SC)**	6	8.839	1.103	25	0
	**6000g/ton (SK)**	9	12.5	3.122	>50	1
	**8000g/ton (NK)**	5	25	25	35.36	0

		MIC: Geometric Mean of 2 Isolates				
		**Number of Animals**	**Median**	**Min**	**Max**	**Number of Isolates >50**
**Oct**	**0g/ton (G)**	6	2.666	1.56	3.125	0
	**700g/ton (TH)**	7	12.5	6.25	50	0
	**5000g/ton (SC)**	8	3.125	2.208	25	0
	**6000g/ton (SK)**	8	12.5	3.125	25	0
	**8000g/ton (NK)**	2	25	25	25	0

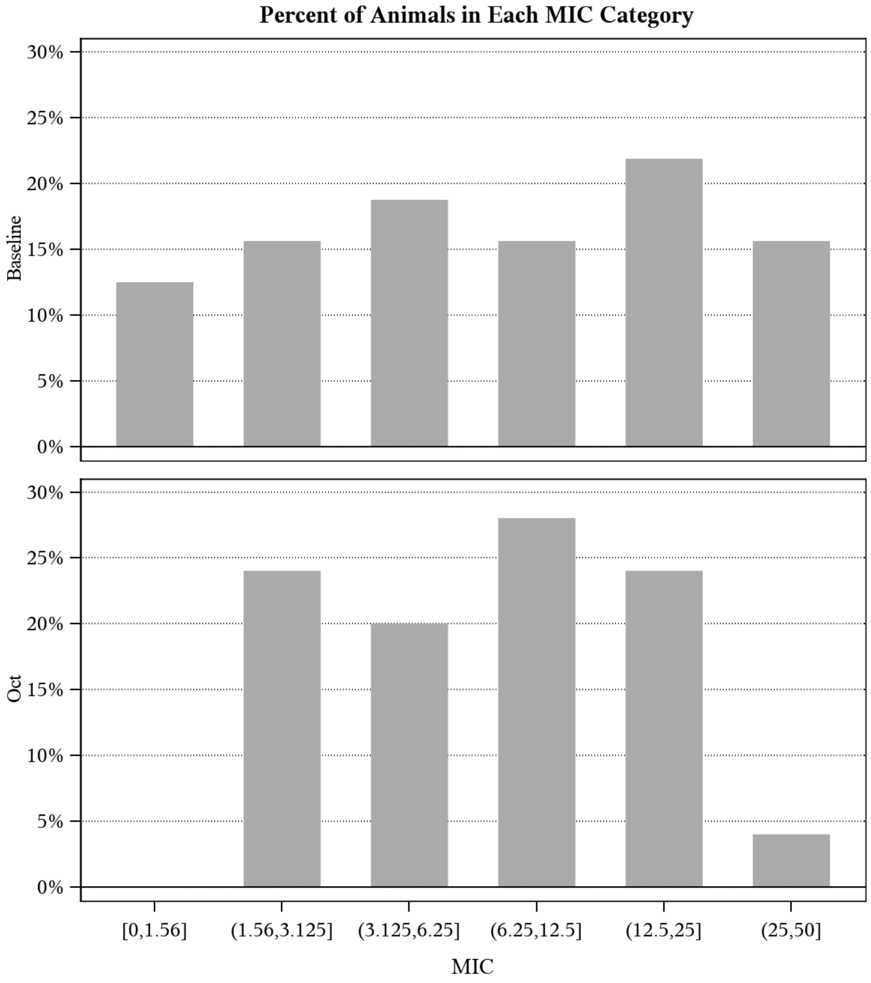

MIC refers to the geometric mean of MICs for two isolates

Supplemental Figure S1. Distribution of MIC values from *E. coli* isolates derived from study groups provided a CTC-medicated feed product at Baseline (prior to CTC exposure) and October (5 months continuous treatment). This data was derived from a subset of the animals that had a MIC value available at baseline or the final sampling time point (Baseline: 32 animals, October: 25 animals). CLSI breakpoints for *E. coli* susceptible (≤4 µg/mL), intermediate (8 µg/ml), or resistant (≥16 µg/mL) to CTC.

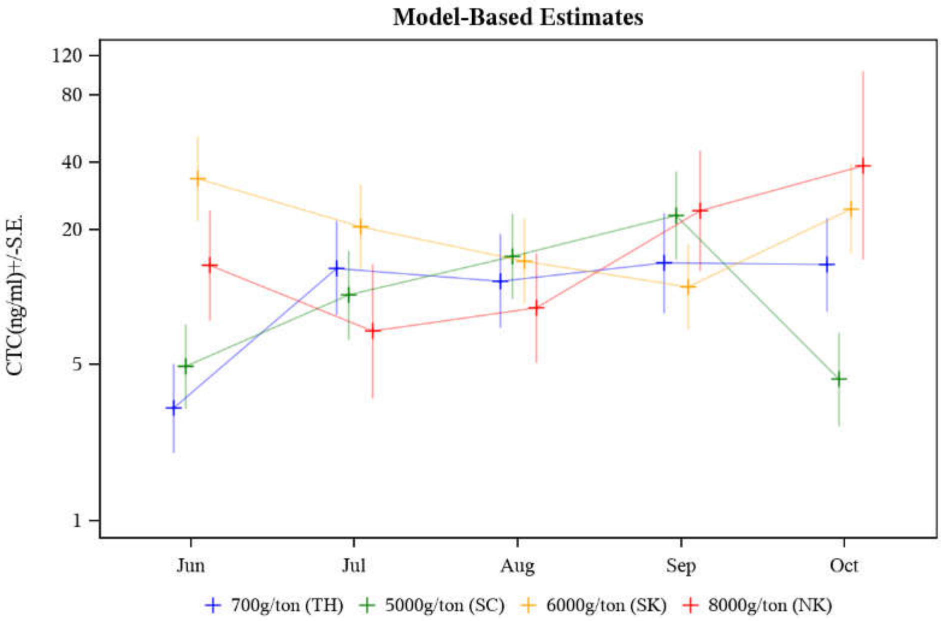

Supplemental Figure S2. Model-based estimates of median plasma CTC values in each treatment group over the treatment period.
